# Multifaceted Benefits of GDF11 Treatment in Spinal Cord Injury: In Vitro and In Vivo Studies

**DOI:** 10.3390/ijms24010421

**Published:** 2022-12-27

**Authors:** May-Jywan Tsai, Li-Yu Fay, Dann-Ying Liou, Yi Chen, Ya-Tzu Chen, Meng-Jen Lee, Tsung-Hsi Tu, Wen-Cheng Huang, Henrich Cheng

**Affiliations:** 1 Neural Regeneration Laboratory, Department of Neurosurgery, Neurological Institute, Taipei Veterans General Hospital, Taipei 11217, Taiwan; 2 Division of Neural Regeneration and Repair, Neurological Institute, Taipei Veterans General Hospital, Taipei 11217, Taiwan; 3 Department of Medicine, National Yang Ming Chiao Tung University, Taipei 11221, Taiwan; 4 Department of Applied Chemistry, Chaoyang University of Technology, Taichung 41349, Taiwan; 5 Institute of Pharmacology, National Yang Ming Chiao Tung University, Taipei 11221, Taiwan

**Keywords:** GDF11, spinal cord injury, neuroprotection, inflammation, neuronal/glial cultures

## Abstract

Traumatic spinal cord injury (SCI) initiates a series of cellular and molecular events that include both primary and secondary injury cascades. This secondary cascade provides opportunities for the delivery of therapeutic intervention. Growth differentiation factor 11 (GDF11), a member of the transforming growth factor-β (TGF-β) superfamily, regulates various biological processes in mammals. The effects of GDF11 in the nervous system were not fully elucidated. Here, we perform extensive in vitro and in vivo studies to unravel the effects of GDF11 on spinal cord after injury. In vitro culture studies showed that GDF11 increased the survival of both neuronal and oligodendroglial cells but decreased microglial cells. In stressed cultures, GDF11 effectively inhibited LPS stimulation and also protected neurons from ischemic damage. Intravenous GDF11 administration to rat after eliciting SCI significantly improved hindlimb functional restoration of SCI rats. Reduced neuronal connectivity was evident at 6 weeks post-injury and these deficits were markedly attenuated by GDF11 treatment. Furthermore, SCI-associated oligodendroglial alteration were more preserved by GDF11 treatment. Taken together, GDF11 infusion via intravenous route to SCI rats is beneficial, facilitating its therapeutic application in the future.

## 1. Introduction

Acute spinal cord injury (SCI) is an unexpected, devastating event that leads to irreversible loss of neurological function [1,2,3]. Trauma to the spinal cord causes direct damage at the lesion site, followed by a secondary degenerative process. The secondary injury occurs over the time course of minutes to weeks. Intervention to block secondary pathological cascade after injury may limit the extent of tissue injury and the consequent disability. Following SCI, damage occurs in both nervous tissue and the surrounding vasculature, which leads to intraspinal hemorrhage [4,5,6] and deficits in tissue perfusion [7]. The damaged adult spinal cord has limited capacity to repair because CNS neurons have a poor intrinsic capacity for growth, but also because injured axons encounter a series of inhibitory factors that are non-permissive for growth. Applying factor with neuroprotective/neuroregenerative functions to intervene damage cascade would be a promising strategy for severe SCI.

Growth differentiation factor 11 (GDF11) regulates various biological processes in mammals. GDF11, a member of the BMP/TGFbeta superfamily, binds to activin receptor I and II and regulates expression of its target genes [8]. GDF11 was identified as a rejuvenation factor that is capable of reversing age-related dysfunction of stem cells and neurogenic function [9,10,11]. However, a controversial effect of GDF11, protective or detrimental, has been reported in age-related diseases. Higher levels of GDF11 are closely associated with a lower risk of cardiovascular events, as reported by Olson et al. [12]. This indicates cardioprotective effects of GDF11. However, other studies have demonstrated that GDF11 might inhibit skeletal muscle regeneration and have no effect on cardiac hypertrophy [13,14]. Kim et al. demonstrated that GDF11 does not affect proliferation of progenitors in developing retina [15], but other studies have demonstrated beneficial effects of GDF11 in modulating synaptogenesis, and improving neurovascular and nerve function in elderly individuals [9,16,17].

The level of GDF11 declines with age, and GDF11supplementation can reverse age-related dysfunction in the brain, heart, and skeletal muscle [9,10,11]. GDF11 treatment was shown to reduce inflammation, oxidative stress, and apoptosis in an experimental intra-cerebral hemorrhage model in old rats [18]. Recent studies have also highlighted that GDF11 has neuroprotective and neurorestorative effects in cerebral ischemic injury [19,20,21]. Lentivirus-mediated GDF11 overexpression in vivo can protect injured nerves, promote axonal growth, and inhibit neuronal apoptosis in the spinal cord [22]. However, a report that GDF11 causes neurotoxicity during ischemia in vitro led to controversy [23]. The underlying mechanisms of GDF11 in the nervous system were not clearly established in previous studies. Here, we perform in vitro and in vivo studies to unravel the effects of recombinant GDF11 on the spinal cord after injury. First, we extensively investigate the effects of GDF11 on neuron–glial cultures in vitro to reveal the underlying mechanism of action. We further examine and discuss the potential effects of GDF11, via intravenous injection, on injured spinal cords in rats.

## 2. Results

### 2.1. Effect of GDF11 Treatment on Cell Survival or Migration

**Figure 1:**
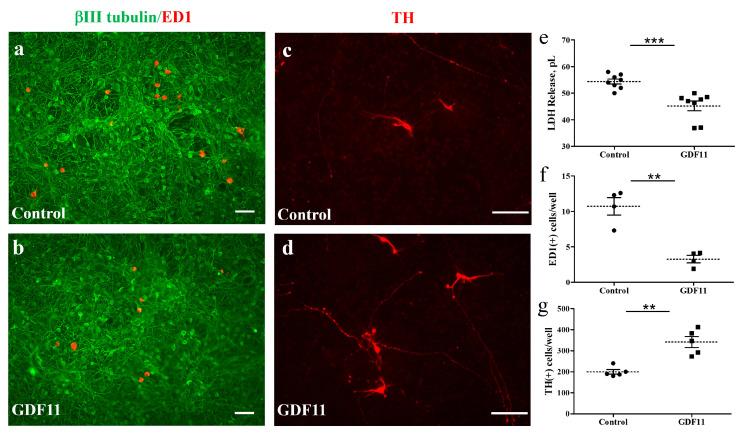
GDF11 enhanced neuronal survival but reduced microglial numbers in neuron–glial cultures. (**a**,**c**) Control cells; (**b**,**d**) GDF11-treated cells. Cells in (**a**,**b**) are immunoreactive (IR) for βIII tubulin (in green) and for ED-1 (in red) and are neurons and microglia, respectively. Magnification: 100×. Cells in (**c**,**d**) are tyrosine hydroxylase (TH)-IR dopaminergic neurons. Magnification: 200×. (**e**) Quantification of lactate dehydrogenase (LDH) release in the culture medium of (**a**,**b**). (**f**) Quantification of ED-1-IR microglia per 0.01516 cm^2^ in the cultures of (**a**,**b**). (**g**) Quantification of TH-IR cell numbers per well (1.131 cm^2^) in the cultures of (**c**,**d**) from representative images. The results are reported as mean ± SEM in each group from three to four independent experiments. ** *p* < 0.01, *** *p* < 0.001 Control versus GDF11 treatment, by unpaired Student *t*-test. Magnification: 100× in (**a**,**b**) and 200× in (**c**,**d**). Scale bar: 100 μm.

The neuron–glial cultures used in the present study were prepared from cortical, mesencephalic, or spinal regions of embryonic rats. We first examined the effects of GDF11 in primary neuron–glial cultures from cortical or mesencephalic regions. On the second day after cell seeding, the cultures were treated with saline or GDF11, 240 ng/mL, for 2 days. After treatment, the cultured medium was saved for lactate dehydrogenase (LDH) assay and the cultures were processed for double-labeled immunostaining with anti-βIII-tubulin, a neuronal marker, and anti-ED1, a microglia marker. Figure 1 shows that LDH levels in the medium, due to cell damage and thereby release of cytosolic LDH into the medium, were markedly reduced by GDF11 treatment (Figure 1e, *p* < 0.001). Because more than 85% of the cells were neuronal cells in the neuron–glial cultures, a reduction in LDH release by GDF11 treatment indicates the effect of increased neuronal survival. By contrast, GDF11 selectively reduced microglial numbers, shown in Figure 1a,b,f, in cortical neuron–glial cultures. Figure 1c,d,g shows that TH-positive dopaminergic neurons were also increased by GDF11 treatment in mesencephalic cultures. These results suggest that GDF11 effectively enhances neuronal survival, but reduces microglial numbers in neuron–glial cultures. The effects of GDF11 were further examined in spinal cord neuron–glial cultures. Two days after saline or GDF11 treatment, the cultures were immunostained with anti-βIII-tubulin and anti-RIP, an oligodendroglial marker probing for 2′,3′-cyclic nucleotide 3′-phosphodiesterase.

**Figure 2:**
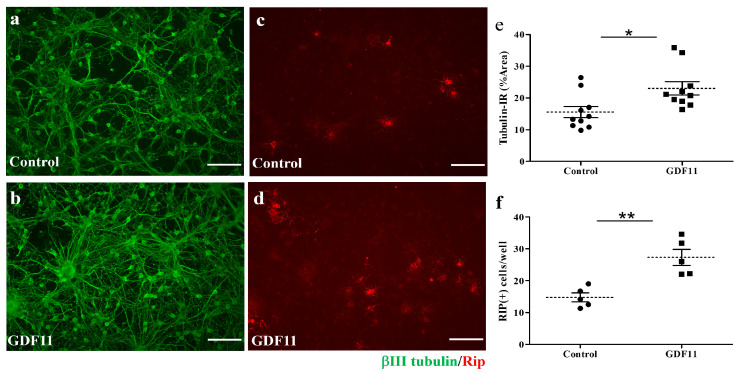
Effect of GDF11 on neuronal or oligodendroglial survivals in neuron–glial cultures. (**a**,**c**) Control cells; (**b**,**d**) GDF11-treated cells. Cells in (**a**,**b**) are βIII tubulin-immunoreactive (IR) neuronal cells. Cells in (**c**,**d**) are Rip-IR oligodendroglial cells. (**e**) Quantification of βIII tubulin-IR cell density in cultures of (**a**,**b**) per 0.00356 cm^2^. (**f**) Quantification of RIP-IR cell numbers in cultures of (**c**,**d**) per 0.00356 cm^2^. The results are reported as mean ± SEM in each group. * *p* < 0.05, ** *p* < 0.01 Control versus GDF11 treatment by unpaired Student *t*-test. Magnification: 200×. Scale bar: 100 μm.

Compared to the control cultures, GDF11 markedly increased tubulin-IR neuronal connection, as shown in Figure 2a,b,e. Concurrently, GDF11 significantly increased oligodendroglial cell numbers (Figure 2c–f). We also found that GDF11 had an effect on neural stem cell (neurosphere) differentiation. As shown in Supplementary Figure S1, GDF11 obviously enhanced the differentiation of neural stem cells into RIP-positive oligodendroglia.

**Figure 3:**
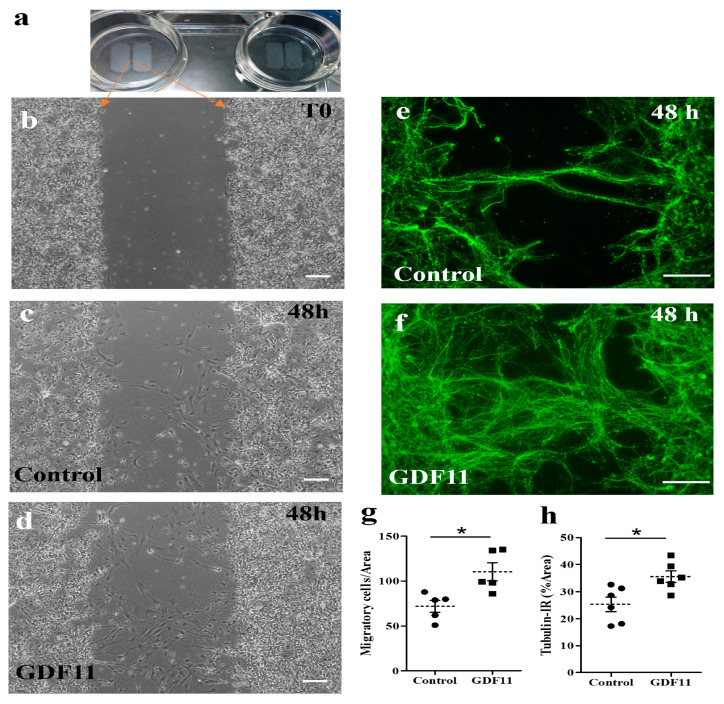
Effects of GDF11 on neurite extension and cell migration in spinal cord neuron–glial cultures. (**a**,**b**) Cells were seeded in ibidi inserts with a gap of approximately 594.2 ± 10.7 μm. After removing the insert and refilling with a new medium, the seeded cells (at T0, shown in (**b**) started to migrate or extend neurite. (**c**,**d**) Representative phase contrast images of control and GDF11-treated cultures, respectively, after a 48 h incubation. Magnification: 100× in Panels (**b**–**d**). (**e**,**f**) Representative βIII tubulin-IR images of control or GDF11-treated cultures, respectively, after a 48 h incubation. Magnification: 200×. (**g**) Quantification of migratory cells (%) in the cultures of (**c**,**d**) by counting migratory cells per gap area of 632,016 μm^2^ at 10× objective. (**h**) Quantification of βIII tubulin-IR neurite density (%) by Image J in the cultures of (**e**,**f**) per gap area of 289,940 μm^2^ at 20× objective. The results are reported as mean ± SEM in each group from three independent studies. * *p* < 0.05 Control versus GDF11 treatments by unpaired Student *t*-test. Scale bar: 100 μm.

To explore the influence of GDF11 on the growth of neuronal axons and cell migration, spinal cord neuron–glial cultures were also seeded in ibidi inserts in 4-well plates. There was a gap of approximately 500 μm between two insert wells. Several hours after cell seeding, the insert was removed and the cultured cells were refilled with fresh medium (DMEM + 4% FCS) in the presence or absence of GDF11 240 ng/mL. The seeded cells (at T0 shown in Figure 3a,b) started to migrate or extend neurites actively. Neuron–glial cultures were incubated for a total of 2 days. The results showed that GDF11 markedly promoted neurite extension with more tubulin-IR neurites found in the gap than those in control cells (*p* < 0.05; Figure 3e,f,h). The tubulin-IR (% area) are based on a gap area (width × height) of about 289,940 μm^2^ at 20× objective. Concurrently, GDF11 markedly induced cell migration toward the gap (*p* < 0.05; Figure 3c,d,g). The migratory cells were counted in the area of about 632,016 μm^2^ at 10× objective.

### 2.2. Neuroprotective Effects of GDF11 Treatment on OGD- or Endotoxin-Induced Damage

**Figure 4:**
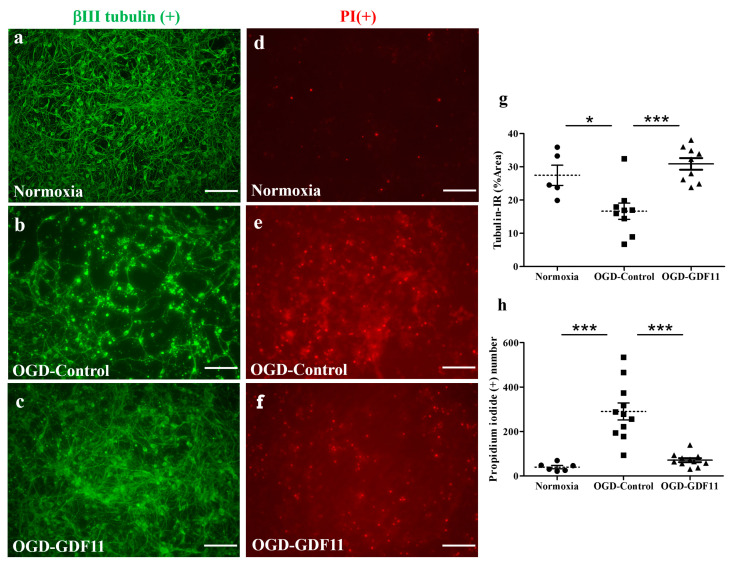
GDF11 protected neuron–glial cultures from oxygen glucose deprivation (OGD)-induced damage. (**a**–**c**) βIII tubulin-IR in normoxia-control, OGD-control, or OGD-GDF11-treated cultures, respectively. (**d**–**f**) Propidium iodide (PI)-positive cells in normoxia-control, OGD-control, or OGD-GDF11-treated cultures. (**g**) Quantification of βIII tubulin-IR neuronal density (%) per 3.56 × 10^−3^ cm^2^ in normoxia and OGD-induced cultures of (**a**–**c**). (**h**) Quantification of PI-positive cells (PI [+] numbers per 3.56 × 10^−3^ cm^2^) in normoxia and OGD-induced cultures of (**d**–**f**). The results are reported as mean ± SEM in each group from three independent experiments. * *p* < 0.05 Normoxia versus OGD-control, *** *p* < 0.001 indicates a significant difference between the OGD control and OGD treatment groups by one-way ANOVA. Magnification: 200×. Scale bar: 100 μm.

Oxygen glucose deprivation (OGD), an in vitro ischemic model, and lipopolysaccharide (LPS) stimulation were employed to examine whether GDF11 was able to protect the stressed cultures. To examine the pharmacological effects of GDF11 on OGD-induced damage, spinal cord neuron–glial cultures first underwent 3 h of OGD stress and were restored to normoxic conditions. Saline or GDF11 240 ng/mL was then added to the cultures, which were further incubated under normoxic conditions for 2 days. OGD-induced cell damage in the spinal cord neuron–glial cultures (Figure 4). GDF11 markedly protected OGD-induced cell damage, as depicted by tubulin-IR or propidium iodide (PI) positive cells in Figure 4b,c,e,f. The densities of tubulin-immunoreactive cells were significantly higher in GDF11-treated cultures compared to those in control cultures after OGD-induced damage (Figure 4b,c,g). Consistently, the numbers of OGD-induced PI-positive cells were markedly attenuated by GDF11 treatment (Figure 4e,f,h).

**Figure 5:**
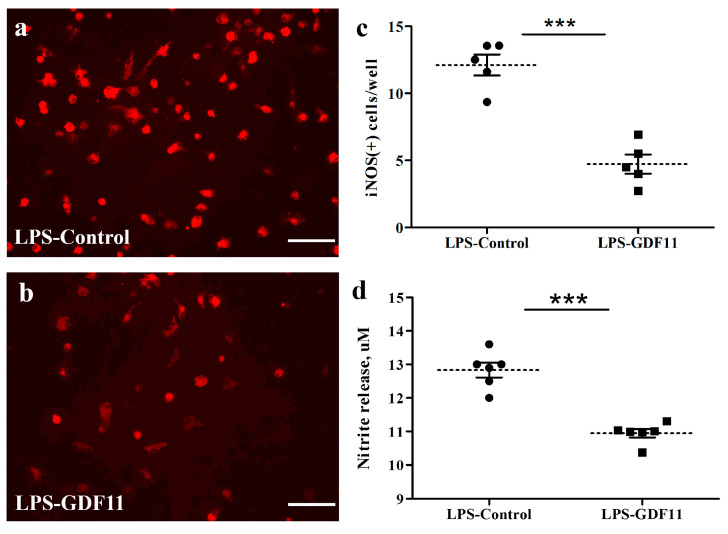
GDF11 reduced LPS stimulation in neuron–glial cultures. (**a**,**b**) iNOS-IR cells in control or GDF11-treated LPS-stimulated cultures, respectively. (**c**) Quantification of iNOS-IR cells (iNOS (+) numbers per 3.56 × 10^−3^ cm^2^) in LPS- stimulated cultures of (**a**,**b**). Notably, GDF11-treated cultures significantly reduced LPS-induced iNOS (+) numbers (*p* < 0.001). (**d**) LPS treatment induced nitrite release to medium in control or GDF11-treated cultures. GDF11-treated cultures significantly reduced LPS-induced nitrite release into the medium (*p* < 0.001). The results are reported as mean ± SEM in each group from three independent experiments. *** *p* < 0.001 indicates a significant difference between the LPS control and LPS treatment groups using unpaired Student *t*-test. Magnification: 200×. Scale bar: 100 μm.

To examine the effect of GDF11 on LPS stimulation, the cultures were switched to a serum-reduced medium (DMEM + 4% FCS). LPS with or without GDF11 was added to the cultures. Two days later, the culture medium was collected for nitrite assay and the cells were processed for immunostaining probing for inducible nitric oxide synthase (iNOS). As shown in Figure 5, GDF11 markedly attenuated the LPS-induced increase in iNOS-IR number and the level of nitrite release to the medium (*p* < 0.05).

**Figure 6:**
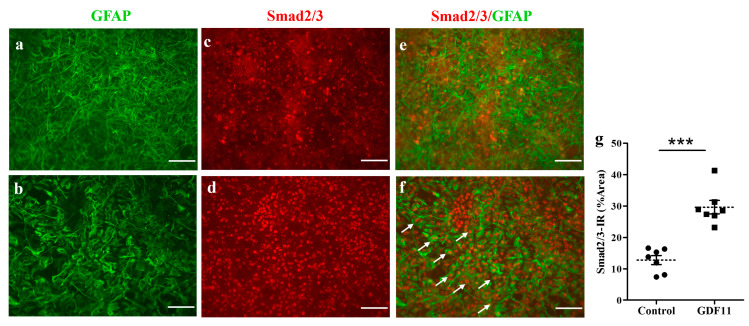
GDF11 induced Smad2/3 translocation in neuron–glial cultures. (**a**,**c**,**e**) Control; (**b**,**d**,**f**) GDF11 2 h treatment; (**g**) The quantitative results of smad2/3 immunoreactivity (IR) in control and GDF11 cultures treated for 2 h; (**a**,**b**): GFAP-IR, (**c**,**d**): Smad2/3-IR, (**e**,**f**): Double-labeling immunoreactivity of GFAP (green) and Smad2/3 (red). The arrows depict nuclear location of Smad2/3 in GFAP-IR astroglia in neuron–glial cultures. The results are reported as mean ± SEM in each group from three independent experiments. *** *p* < 0.001 indicates a significant difference between the control and GDF11 treatment by unpaired Student *t*-test. Magnification: 200×. Scale bar: 100 μm.

GDF11 is a member of the activin subfamily, which is also classified as a new member of the TGF-β superfamily, capable of activating the Smad2/3 signaling pathway [24]. To explore the pathway by which GDF11 acts, we measured Smad2/3 immunoreactivity in neuron–glial cultures 2 h after GDF11 treatment. Interestingly, GDF11 markedly induced nuclear translocation of Smad2/3 in the cultured cells, with some of Smad2/3-IR being in astroglia, after a 2 h treatment (*p* < 0.001; Figure 6).

### 2.3. Intravenous GDF11 Treatment Preserved Spinal Cord Tissues and Promoted Functional Recovery

**Figure 7:**
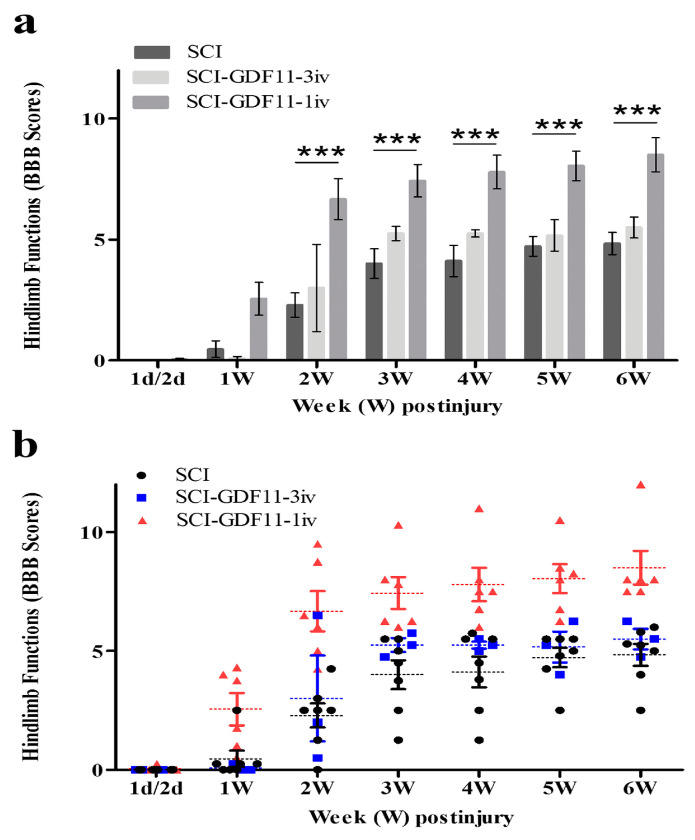
Time course of hindlimb locomotor recovery in rats receiving saline or GDF11 after contusive injury. (**a**) Data are plotted as a bar chart. (**b**) Data are plotted with individual data points. Within 30 min of injury, SCI rats were injected with saline or GDF11 through the tail vein once (24 μg/rat) or for 3 consecutive days (8 μg/rat/injection, daily). Locomotor recovery of the SCI rats was evaluated over a 6-week period, using a 21-point scale (Basso–Beattie–Bresnahan (BBB) locomotor rating scale). The hindlimb recovery of the animals was assessed in a double-blind manner. The results are reported as mean ± SEM from 7, 3, and 6 rats for SCI, SCI-GDF11-3iv, and SCI-GDF11-1iv, respectively. Statistical significance was evaluated using two-way ANOVA and Bonferroni’s *t*-test. *** *p* < 0.001.

To study the in vivo effect of GDF11, we used a severely contusive SCI rat model. Within 30 min of eliciting a contusive injury on the spinal cord of a rat, GDF11 was administered to the SCI rat through tail vein infusion. Intravenous administration of one bolus of GDF11, 24 μg/rat, to the rats with contusive spinal cord injury showed beneficial effects in SCI rats (Figure 7). Beginning two weeks post injury and throughout the experimental periods, GDF11 treatment effectively improved the hindlimb behaviors (BBB scores) of the SCI rats (Figure 7a,b). However, lower doses of GDF11 treatment, daily 8 μg injection for three consecutive days, did not influence the functional restoration of SCI rats.

**Figure 8:**
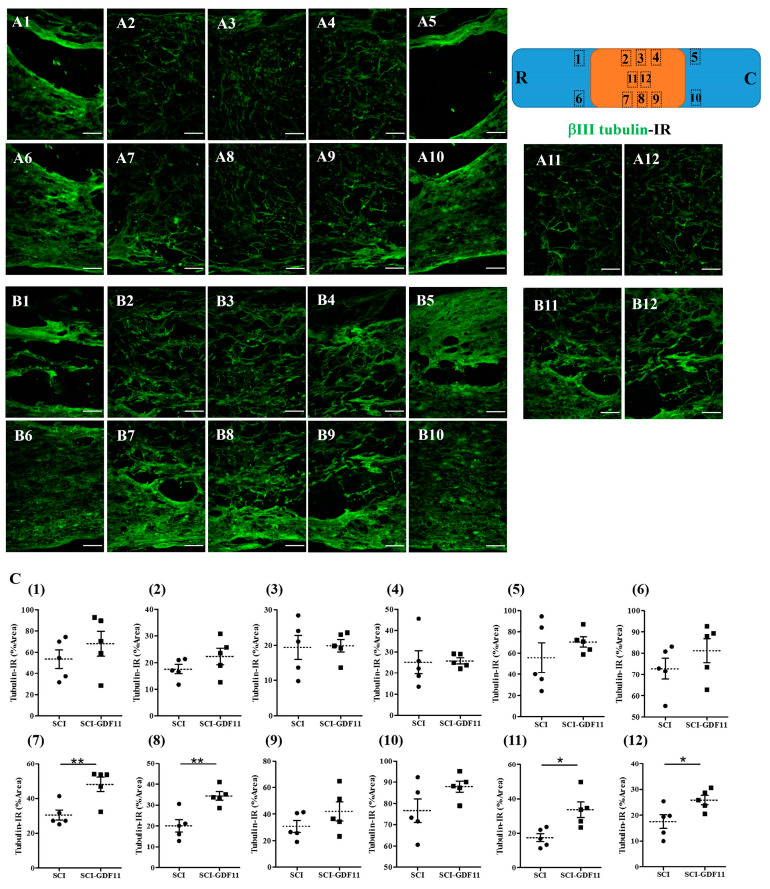
βIII Tubulin-immunoreactive nerve fibers in the thoracic spinal cords of SCI rats 6 weeks after injury. (**A**) Representative images of longitudinal spinal cord sections in an SCI rat. (**B**) Representative images of spinal cord sections in a GDF11-treated SCI rat. (**C**) Quantification of βIII tubulin-positive axons shown in area (1–12) of (**A**) or (**B**) panel, from control- or GDF11-treated, respectively, in spinal cord sections. The upper-right scheme shows segments of the thoracic spinal cord (T7~T9; R: rostral; C: caudal). The locations of tubulin-IR photo taken in area (1–12) are depicted. Note the significant increase in tubulin-positive nerve fibers in the GDF11-treated groups in areas (7), (8), (11), and (12) at 6 weeks post-injury. GDF11, 24 μg/rat, was administered to SCI rats through tail vein injection after spinal cord contusion. After 6 weeks, immuno-fluorescence positive areas of βIII tubulin were detected in the longitudinal sections at the epicenter of the spinal cord. The results are reported as mean ± SEM from 5 rats per group. Statistical significance is evaluated using unpaired Student *t*-test. * *p* < 0.05, ** *p* < 0.01, respectively, SCI versus SCI-GDF11. N = 5 rats per group. Magnification: 200×. Scale bar: 100 μm.

Six weeks after injury, the SCI rats with or without treatment were sacrificed. Their spinal cords were removed and processed for histological analysis. Selected spinal cord sections were immunostained with anti-βIII tubulin or anti-RIP and with proper secondary antibodies. Figure 8 shows that a few βIII tubulin-IR fibers could be observed in the epicenter of thoracic spinal cords. GDF11-treated spinal cord sections preserved more βIII tubulin-IR fibers in the epicenter than those in an SCI rat spinal cord. Specifically, βIII tubulin-IR in the median and ventral parts of the spinal cord (regions B7, 8, 11, and 12) was better preserved by GDF11 treatment.

**Figure 9:**
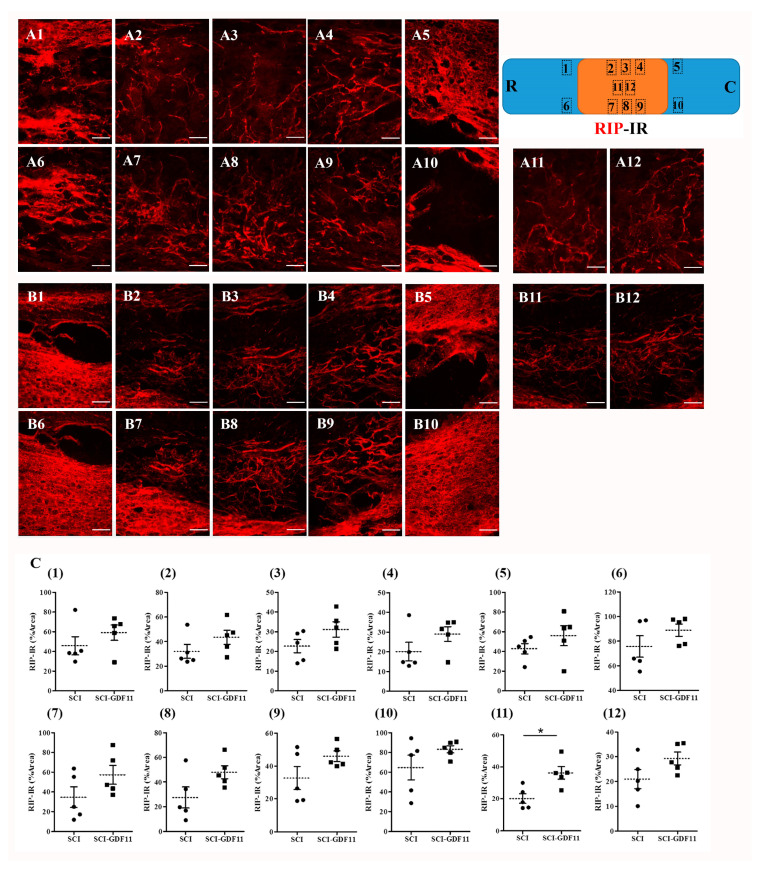
RIP-immunoreactive oligodendroglia in the thoracic spinal cords of SCI rats 6 weeks after injury. (**A**) Representative images of longitudinal spinal cord sections in an SCI rat. (**B**) Representative images of spinal cord sections in a GDF11-treated SCI rat. (**C**) Quantification of RIP-IR shown in areas (1–12) of (**A**) or (**B**) panel from, respectively, control- or GDF11-treated spinal cord sections. The upper-right scheme shows segments of the thoracic spinal cord (T7~T9; R: rostral; C: caudal). The locations of RIP-IR photo taken in areas (1–12) were depicted. Note the significant increase in RIP-positive oligodendroglia in GDF11-treated groups in area (11) at 6 weeks post-injury. RIP-IR in area (8) has a tendency to increase (*p* = 0.077) but did not reach a significant level. GDF11 was administered to SCI rats through tail vein injection after spinal cord contusion. After 6 weeks, immunofluorescence-positive areas of RIP were detected in a longitudinal section at the epicenter of the spinal cord. The results are reported as mean ± SEM from 5 rats per group. Statistical significance is evaluated using unpaired Student *t*-test. * *p* < 0.05 SCI versus SCI-GDF11. Magnification: 200×. Scale bar: 100 μm.

To see the effect of GDF11 treatment on oligodendroglial cells in SCI rats, spinal cord sections were immunostained by anti-RIP probing for oligodendroglial cells. Figure 9 shows RIP-IR in the epicenter of the spinal cord in SCI rats at 6 weeks post-injury. Figure 9C shows the quantification of RIP-IR, shown in area 1–12 of A or B panel, from control- or GDF11-treated spinal cord sections. Note the significant increase in RIP-positive oligodendroglia in the GDF11-treated groups in area 11 at 6 weeks post-injury. RIP-IR in area 8 had a tendency to increase but did not reach a significant level (*p* = 0.077).

## 3. Discussion

Spinal cord injuries and neuropathy are prevalent in clinical practice. However, treatment is limited in terms of minimizing secondary complications. The present work performs extensive in vitro and in vivo studies to unravel the effects of a rejuvenation factor GDF11 on an injured spinal cord. We present evidence supporting the notion that GDF11 effectively increases cell survival and reduces the extent of spinal cord neuronal injury, both in vivo and in vitro.

The beneficial effects of GDF11 were first demonstrated in primary neuron–glial cultures. GDF11 treatment effectively promoted neurite extension and enhanced the survival of both neuronal and oligodendroglial cells but reduced microglial numbers (Figure 1, Figure 2 and Figure 3). Furthermore, GDF11 could protect neuron–glial cells from OGD-induced injury and LPS stimulation (Figure 4 and Figure 5). The in vivo study further demonstrated that one bolus of GDF11 infusion during the acute stage of injury possessed therapeutic potential for the treatment of traumatic SCI. GDF11 treatment not only protected spinal cord tissues but significantly enhanced hindlimb behavior restoration in SCI rats. Supporting the observed behavioral improvements, the nerve fibers (axons) and oligodendroglial cells (myelins) were more preserved in the GDF11-treated spinal cords, as shown in Figure 7, Figure 8 and Figure 9.

The protective effects of GDF11 in cultures as well as in SCI rats are consistent with previous studies, showing that GDF11 benefits cerebral ischemic injury [19,20,21] and spinal cord injury [22]. Besides, the present study highlighted the merits of GDF11 to oligodendroglia in both neuron–glial cultures (Figure 2) and neural stem cells (Supplementary Figure S1). GDF11 not only increased oligodendroglial survival in neuron–glial cultures, but also induced oligodendroglial differentiation from neural stem cells. The in vivo study with intravenous GDF11 infusion in SCI rats also preserved oligodendroglial cells in the injured spinal cord (Figure 9). These results were novel and interesting, deserving further studies. The present study also demonstrated that GDF11 plays a role in reducing microglial numbers in neuron–glial cultures. Because a nuclear translocation of Smad2/3 was found at an early stage, 1–2 h, after GDF11 treatment (Figure 6), this observed effect in microglia could be possibly mediated through Smad2/3 or non-Smad signaling. The underlying mechanism of GDF11 on microglial numbers remains to be determined. In Figure 5, the LPS-stimulated iNOS expression was attenuated significantly by GDF11 in neuron–glial cultures. Hsu and Wen [25] suggested that the LPS-induced generation of reactive oxygen species in microglia/macrophages is an upstream event serving to regulate the production of other pro-inflammatory factors. Reducing activated microglial numbers available in cultures by GDF11 would provide neuronal protection against LPS stimulating cascade.

Wang et al. [26] demonstrated that GDF11 induces apoptosis and suppresses the migration of C17.2 stem cells. A recent report from the same group [27] also showed that GDF11 significantly suppresses the cell proliferation/migration and promotes the differentiation/apoptosis of pheochromocytoma cells (PC12). Studies by Wang and coworkers employed two kinds of cell line, i.e., C17.2, an immortalized neural progenitor cell line, and pheochromocytoma PC12 of the rat adrenal medulla. In contrast to the results reported by Wang and coworkers (2018 and 2022), our results demonstrate that GDF11 treatment markedly increases both cell migration and neurite outgrowth (Figure 3). This result is in line with the report of Katsimpardi et al. [9], who found that the circulatory GDF11 in blood promotes migration of neural cells. Cell lines are immortalized cells with some changed properties, different from those of the original cells. Furthermore, PC12 originated from the cells of the adrenal medulla, which are not even in the nervous system. The present study used primary cultures from fetal brain or spinal cords, which were used within one week of cell seeding. The cell characteristics altered a little and were much more similar to their in vivo counterpart. Therefore, the results of Wang et al. [26,27] cannot be simply compared with our results.

Intriguingly, Sutherland et al. [23] demonstrated the neurotoxic effect of GDF11 during ischemia in vitro (OGD). However, GDF-11 treatment during the 24 h recovery period after 2 h OGD had no effect oncell survivals. Sutherland et al. found that GDF-11 neurotoxicity occurred following neuronal exposure to hydrogen peroxide [23]. The protective GDF11 treatment used in the cell cultures of the present study does not differ from that of Sutherland et al. [23], since GDF11 was added to cultures only after OGD insult and during recovery. However, we also observed the protective effect of GDF11 on peroxide toxicity in cultures (our unpublished results). The different results obtained after GDF 11 treatment can be attributed to lot-to-lot variability in the commercially available recombinant GDF 11, and to the fact that the dosage of GDF11used is different. In the present study, cultured cells were exposed to a higher concentration of GDF11, 240 ng/mL. Furthermore, the stock solution of GDF11 was carefully neutralized before use in cell cultures or in SCI rats.

Previous studies have shown that the TGF family, including GDF11, plays a role in the Smad signaling pathway [28,29]. Lin et al. [10] pointed out that GDF11 is a member of the activin subfamily, capable of activating the Smad2/3 signaling pathway. The Smad protein complex then translocates into the nucleus and interacts with transcriptional co-activators or co-repressors to regulate promoter activity in control gene expression. To explore the pathway by which GDF11 acts, we measure Smad2/3 and the phosphorylation form of Smad2/3 in cultures at different time points after GDF11 treatment. We could not observe any difference in the levels of phosphor-Smad2/3 by GDF11 treatment. Interestingly, we found that GDF11 induces nuclear translocation of Smad2/3 in neuron–glial cultures after a 2 h treatment (Figure 6). Furthermore, some of nuclear Smad2/3-IR was localized in GFAP-positive astrocytes (arrows, Figure 6f). Apparently, changes in the astroglial morphology were concurrently observed, and both phenomena were almost back to normal 48 h after treatment (Figure 6). Our results are consistent with previous studies, showing that GDF11 may play a role in the Smad2/3 signaling pathway [29,30]. The receptor and specific mechanism of GDF11 in neurons should be further explored in future studies. Whether the protective/anti-inflammatory effects of GDF11 observed in the present study are mediated through the Smad signaling pathway requires further studies, too.

GDF11 was applied to aged mice through peripheral iv or ip routes, resulting in significant neuronal/vascular improvement in the brains [31]. Ozek and coworkers employed various approaches to prove that GDF11 action is on brain vasculature, rather than on crossing the blood brain barrier (BBB) [31]. It is known that the BBB disruption occur hours after contusive spinal cord injury, but it would recover several days later [32]. In the present study, GDF11 was administered intravenously to SCI rats at the acute stage after eliciting a contusive injury on their spinal cord. GDF11 may have a direct or indirect effect on the injured cord and improve functional restoration. Even small gains from neuroprotection can have significant functional effects following SCI. The SCI rats with GDF11 iv infusion exhibited better functional recovery than the control group, indicating that the spinal cord of the GDF11 group was less severe than that of the control group. The GDF11 group consistently had more preserved βIII tubulin labeling of spinal nerve fibers. We also used antibody RIP to label myelin-forming oligodendroglia, confirming that GDF11 could indeed benefit spinal nerve fibers by promoting the formation or preservation of extraaxonal myelin structures. Our results are in agreement with the reports of Lin et al. [30], who found that GDF11 inhibits neuronal damage both in vivo and in vitro, and that the corresponding number of neurons in the spinal cord segment was higher.

In conclusion, the present work discovered that GDF11 exhibits neuroprotective and neurorestorative effects on the stressed spinal cord. Cell culture studies highlighted that GDF11 not only enhances both neuronal and oligodendroglial survival but protects cells from ischemia or inflammatory stimulation. In vivo studies further demonstrated that GDF11 treatment benefits SCI rats and might provide an environment that is more conducive to corticospinal axonal regrowth after spinal cord injury.

## 4. Materials and Methods

### 4.1. Materials

Cultured medium, serum-free supplements, and antibiotics were purchased from Invitrogen (Thermo Fisher Scientific, Grand Island, NY, USA). Fetal calf serum (FCS) was from Hyclone (Hyclone Laboratories Inc., Logan, UT, USA). Human recombinant GDF11 was purchased from Peprotech (Rocky Hill, NJ, USA). The lyophilized GDF11 powder was first dissolved in 1N hydrochloric acid-PBS solution and carefully neutralized by 1N sodium hydroxide. Tissue culture plastics were from BD Bioscience. Primary antibodies and suppliers were as follows: rabbit anti-neuronal class III beta tubulin (BioLegend, San Diego, CA. USA), mouse anti-ED1 (Bio-Rad, Hercules, CA, USA), etc. Unless stated otherwise, all other chemicals/reagents were purchased from Sigma-Aldrich (Louis, MO, USA).

### 4.2. Neuron–Glial Cultures and Treatment

Mixed neuronal/glial cell cultures were prepared from embryonic Sprague–Dawley (SD) rats at gestation days 15–17 as described in our published articles [33,34]. Briefly, cortical, mesencephalic, or spinal cord regions of fetal tissues were dissected and dissociated with mixtures of papain/protease/deoxyribonuclease I (0.1%:0.1%:0.03%). The dissociated cells were plated onto poly-D-lysine coated dishes at a density of 1−2 × 10^5^ cells/cm^2^ and maintained in Dulbecco’s modified Eagle’s medium (DMEM) supplemented with 10% FCS. Cultures were incubated at 37 °C in a chamber with a water-saturated atmosphere of 5% CO_2_/95% air. The cultures were treated on the second day after seeding and harvested 2–3 days after treatment. For oxygen glucose deprivation (OGD) treatment, the cultures were washed thoroughly with three changes of PBS, and the media was replaced with glucose-free DMEM. The culture was then placed into an airtight chamber modulated by a ProOx 110 Oxygen Controller (BioSpherix, Redfield, NY, USA) to obtain 0.5% O_2_ with a gas mixture of 5% CO_2_/95% N_2_. After 3 h of OGD, reperfusion was simulated by replacing the exposure medium with normal growth medium. The culture was then treated with GDF11 immediately after OGD-reperfusion or normoxic treatment. One or two days after the OGD treatment, the cultures were harvested for both viability and neurite density measurement. Propidium iodide was applied to the cultures at a concentration of 1 μg/mL 4 h before cell fixation. For LPS treatment, the culture medium was switched to DMEM + 4% FCS, and 10 μg/mL LPS, in the presence or absence of GDF11, was added to the medium. Two days later, the culture medium was collected for a nitrite assay, while cells were processed for double-labeled immunostaining with antibodies against ED1 (microglial marker) or inducible nitric oxide synthase (iNOS). To see the effect of treatment on cell migration or neurite outgrowth, mixed neuronal/glial cells were seeded in ibidi inserts in a 4-well plate. There was a gap of approximately 500 μm between two ibidi insert wells. After insert removal and refilling the cultured cells with a new medium and FBS in the presence or absence of GDF11, the seeded cells started to migrate and the neurons extended neurites. Two days later, the cultures were processed for immunostaining.

### 4.3. Biochemical Assay

LDH is an enzyme that catalyzes the conversion of lactate to pyruvate. The cell injury was assessed by determining the amount of LDH released into the culture medium, using an LDH assay kit (Promega, Madison, WI, USA). After treatment, the LDH released in the culture supernatants was measured with a 30 min coupled enzymatic assay. LDH activity converts NAD^+^ to NADH, which results in the conversion of a tetrazolium salt (INT) into a red formazan product in conjunction with diaphorase. The amount of color formed is proportional to the number of lysed cells. During the assay, serial dilution of standard LDH in picoliter (pL) was carried out concurrently to make a calibration curve, from which the LDH level in the culture medium could be extrapolated to give a value in pL. The nitrite concentrations in the conditioned medium of primary cultures were assayed using the Griess reagent, as described by Tsai et al. (2007). Briefly, after 2 days of LPS treatment, the culture supernatants (150 μL) were collected and mixed with 50 μL of Griess reagent containing 1% sulfanilaminde/0.1% naphthyl ethylene diamine dihydrochloride/2% phosphoric acid and incubated at room temperature for 10 min. The absorbance was measured at 540 nm. Sodium nitrite (NaNO_2_) was used as the standard to calculate the amount of nitrogen dioxide (NO_2_).

### 4.4. Spinal Cord Contusive Injury Rat Model and Treatment

Contusive spinal cord injury was caused in adult female SD rats (Institutional Animal Care and Use Committee (IACUC 2016-084 and IACUC 2016-291). To induce anesthesia, the experimental rats were exposed to 5% isoflurane/95% oxygen in a plexiglass chamber. After induction of anesthesia, the fur on the back was shaved. The rats were then placed in a stereotaxic frame. During surgery, the anesthetic was reduced to 2.5% isoflurane/oxygen and the rats breathed through the inhalation mask of a vaporizer. Surgery was performed with the help of an operating microscope (Zeiss, Aalen, Germany). After skin incision and dissection of the muscle layers covering the vertebrae, serrate muscles were spread with a retractor. A laminectomy was performed on the thoracic vertebra (T9-T11) without causing any damage to the dura mater. To suspend the spinal cord to cause contusion injury, the spine was immobilized with a vertebral clamp. The impactor rod was positioned centrally at T9/T10 over the spinal cord midline. To inflict a wound on the dorsal surface of the spinal cord, a 10 g rod was dropped from a height of 50 mm. Following this procedure, the rats were released from the clamps. The muscle and skin were closed with interrupted sutures and disinfected with iodine. After spinal cord contusive injury, the rats were assigned to one of following treatment groups: (1) SCI + saline; (2) SCI + GDF11 (PEPROTEC, Rocky Hill, NJ, USA) 8 μg per injection daily for 3 consecutive days; (3) SCI + GDF11 24 μg, one single injection within 30 min of eliciting injury. The dose for a single injection of GDF11 was based on previous studies in which GDF11 was administered to rats at a dose of 0.1 mg/kg [10,35]. Saline or GDF11 was infused through tail vein injection to contusive SCI rats. The animals were then taken out of anesthesia and given 2 × 2.5 mL isotonic saline and antibiotic treatment via subcutaneous injection. Postsurgical care included food pellets soaked in water and a water bottle with longer tube. Bladder evacuation was applied twice daily until the rats could urinate spontaneously. Behavior tests (BBB locomotor scales) of experimental rats were conducted weekly after SCI and treatment. Two months after treatment, the rats were transcardially perfused with PBS, followed by 4% paraformaldehyde. The spinal cords of the rats were carefully removed, dissected, and processed for immunohistochemical staining.

### 4.5. Behavioral Examination

Recovery of hindlimb movements was evaluated using the Basso–Beattie–Bresnahan (BBB) locomotive function test for 5 min in an open field [36]. The rats were adapted to an open field first. The walking pattern of the rats was recorded for 5 min in digital video after they walked continuously in an open field,. The locomotor function was evaluated by two independent investigators who were blinded to the rats’ treatment status. The open field locomotor activity score was determined by observation and scoring of behaviors involving the trunk, tail, and hindlimb. BBB scores range from 0 to 21 (0, no movement; 21, normal movement). Scores of 0 to 7 indicate the return of isolated movements in the three joints (hip, knee and ankle). Scores of 8 to 13 indicate the return of paw placement and coordinated movements with the forelimbs. Hindlimb function was assessed weekly for 6 weeks.

### 4.6. Immunohistochemistry

For in vitro immunocytochemistry assessment, the cultures were fixed in 4% paraformaldehyde for 30 min. Cells were further permeabilized with 0.2% Triton X-100 and blocked with 2% serum. The cultures were subsequently immunostained with primary antibodies, followed by probing with the respective fluorescently tagged secondary antibodies (Jackson ImmunoResearch Inc., West Grove, PA, USA). We used the following primary antibodies, usually in a double staining protocol and the dilutions indicated in parenthesis: Primary antibodies included rabbit anti-βIII tubulin (BioLegend, San Diego, CA, USA; 1/250), mouse anti-GFAP (EMD Millipore corporation, Temecala, CA, USA; 1/250), mouse anti-CD68 (Bio-rad, Hercules, CA, USA; 1/150), mouse anti-RIP (DSHB, Iowa City, IA, USA; 1/150), rabbit anti-iNOS (abcam, Cambridge, MA, USA; 1/150), mouse anti-tyrosine hydroxylase (EMD Millipore corporation, Temecala, CA, USA; 1/150), rabbit anti-Smad2/3 (1/150), or anti-phospho Smad2/3 (1/80) (Cell signaling technology, Danvers, CO, USA). Secondary antibodies were labeled with fluorescent dyes: Donkey anti-rabbit IgG, Cy3 (Jackson; 1/500), and Donkey anti-mouse IgG, Alexa-488 (Jackson; 1/500). Images of the cultured cells were obtained with a fluorescent microscope equipped with fluorescence optics and a CCD camera. Immunocytochemistry of spinal cord sections was performed for in vivo assessments. At the specified post-injury times, the rats received an overdose of pentobarbital and were perfused intravascularly with 0.9% saline and 4% paraformaldehyde in PBS. The spinal cords were collected and set into OTC on the end of the mounting block. The sample was plunged directly into liquid nitrogen for 10–15 s immediately after being secured to the block. The frozen samples were then removed and stored in a −80 °C freezer. Spinal cord samples were sectioned at a thickness of 20 μm in a cryostat. The sections were air-dried and stored. Before staining procedures, the sections were placed in 0.1% triton X-100 for 15 min and 5% serum-containing blocking solution for 30 min. They were processed for incubation with primary antibodies followed by secondary antibodies with a fluorescent tag.

### 4.7. Cell Count Assessment

Because the antibody anti-ED1 probes a macrophage protein localized in the cell membrane, endosome membrane, and lysosome membrane, round-shaped ED1-positive cells were counted in a view, i.e., a photo area of 0.01516 cm^2^ with ×10 objective. For TH-IR, it was not evenly distributed in neuron–glial culture, so that all TH-positive cells were counted in every well. The tubulin-IR density of a view, 0.00356 cm^2^, or a gap area, 0.00289 cm^2^, with 20× objective, was analyzed using Image J program (1.48v, Wayne Rasband, National Institutes of Health, Bethesda, MD, USA). All cell markers were analyzed in two to three culture wells per experiment and three independent experiments per condition. Photographs of rat spinal cord sections were analyzed using Image J program, applying the same brightness/contrast adjustments and threshold values for each marker.

### 4.8. Statistical Analysis

Unless stated otherwise in the figure legends, data are presented as mean values ± standard error of the mean (SEM). Statistical analyses were performed using GraphPad Prism 5.0 (GraphPad Software, San Diego, CA, USA). The significant differences between groups were analyzed using unpaired Student t-test for two groups or one- or two-way analysis of variance (ANOVA), followed by Bonferroni *t*-test for more than two groups, as indicated in the figure legends. A value of *p* < 0.05 was considered statistically significant.

## Data Availability

Not applicable.

## Supplementary Materials

The supporting information can be downloaded at: https://www.mdpi.com/article/10.3390/ijms24010421/s1.
