# Supplementary material for: Multifaceted Benefits of GDF11 Treatment in Spinal Cord Injury: In Vitro and In Vivo Studies
Source: Int J Mol Sci. 2022 Dec 27;24(1):421. doi: 10.3390/ijms24010421 (PMC9820576; doi:10.3390/ijms24010421)
Supplement: Supplementary file 1 [file ijms-24-00421-s001.zip › ijms-2062492-supplementary.pdf]

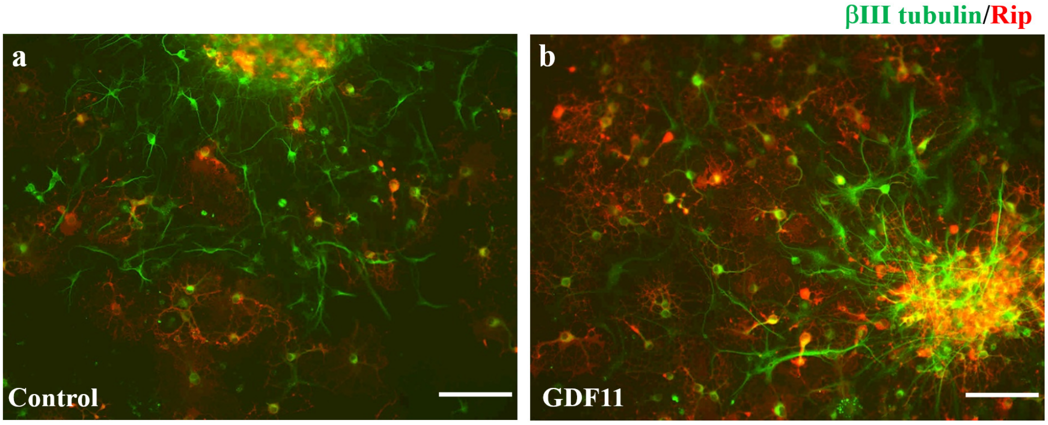

Figure S1. Effect of GDF11 on neuronal or oligodendroglial differentiation from neural stem cells. (**a**) Control; (**b**) GDF11 treatment. Neural stem cells, prepared from embryonic spinal cords, were seeded to poly-D-lysine well in serum-free DMEM medium supplemented with N2 (Gibco). After cell seeding, the medium was replaced with fresh DMEM + N2. GDF11 or saline was added to cultures and incubated for 3–4 days. Anti-βIII tubulin (in green) stains neuronal cells, while anti-RIP (in red) stains oligodendroglial cells. Magnification: 200×.
